# Cullin 3-mediated ubiquitination restricts enterovirus D68 replication and is counteracted by viral protease 3C

**DOI:** 10.1128/jvi.00354-25

**Published:** 2025-05-21

**Authors:** Yan Li, Limei Qu, Yubin Tang, Fushun Ni, Siyu Shen, Haoran Guo, Xiao-Fang Yu, Wei Wei

**Affiliations:** 1 Department of Pathology, The First Bethune Hospital of Jilin University, Changchun, China; 2 Institute of Virology and AIDS Research, First Hospital, Jilin University, Changchun, China; 3 Key Laboratory of Pathobiology, Ministry of Education, Nanomedicine and Translational Research Center, China-Japan Union Hospital of Jilin University, Changchun, China; 4 Cancer Institute (Key Laboratory of Cancer Prevention and Intervention, China National Ministry of Education), The Second Affiliated Hospital, Zhejiang University School of Medicine, Hangzhou, China; 5 Cancer Center, Zhejiang University 12377, Hangzhou, China; 6 Cancer Center Key Laboratory of Organ Regeneration and Transplantation of Ministry of Education, Institute of Translational Medicine, First Hospital, Jilin University, Changchun, China; University of Kentucky College of Medicine, Lexington, Kentucky, USA

**Keywords:** enterovirus D68, Cullin 3, ubiquitination, protein degradation, protein cleavage

## Abstract

Enterovirus D68 (EV-D68) has emerged as a significant threat to public health because of its association with respiratory illnesses and neurological complications, including acute flaccid myelitis. However, the molecular mechanisms underlying EV-D68 replication and pathogenesis remain unclear. Here, we revealed a novel interaction between EV-D68 and the host Cullin-RING E3 ligase system, specifically Cullin 3, which was reported to restrict viral replication. We initially demonstrated that proteasome inhibition enhanced EV-D68 replication, suggesting an important role for the ubiquitin–proteasome system in viral restriction. Cullin 3 was further identified as a key factor that inhibits EV-D68 replication, and the downregulation of its expression increased viral titers. Mechanistically, Cullin 3 was observed to target the viral capsid protein VP1 for ubiquitination and degradation. However, EV-D68 was determined to utilize its protease 3C to cleave Cullin 3 at the Q681 residue, thereby inhibiting E3 ligase activity and facilitating resistance to Cullin 3-mediated VP1 degradation. This study uncovered a host–virus arms race, wherein the ubiquitin–proteasome system of the host actively targets viral proteins for degradation, and viral proteases counteract this defense mechanism. Accordingly, these findings could lead to more effective antiviral treatments.

**IMPORTANCE:**

The ubiquitin–proteasome system (UPS) is a critical cellular pathway involved in the regulation of protein stability and has been implicated in the regulation of viral infections. However, its role in EV-D68 infection has not been extensively explored. Our study proves that the host UPS, through the scaffold protein Cullin 3, can restrict EV-D68 replication, representing a previously unrecognized antiviral mechanism. Furthermore, we describe a viral strategy used to evade this host defense mechanism comprising Cullin 3 cleavage, which has broad implications for understanding virus–host interactions and could inform the development of novel therapeutic strategies against EV-D68 and other enteroviruses.

## INTRODUCTION

Enterovirus D68 (EV-D68), first isolated in 1962, has emerged as a significant pathogen that causes respiratory illnesses and neurological complications, including acute flaccid myelitis (AFM), particularly in children. Initially considered a rare pathogen, EV-D68 gained notoriety in 2014 when it was linked to an AFM outbreak in the United States, highlighting its potential to cause severe health issues (1). The periodic nature of EV-D68 outbreaks, with a peak in transmission typically occurring every 2 years between August and November, underscores the importance of understanding the molecular mechanisms underlying its pathogenesis to develop effective countermeasures.

The ubiquitin–proteasome system (UPS) is a critical pathway involved in regulating protein stability and functions in eukaryotic cells (2). This system tags proteins with ubiquitin, a small protein that is highly conserved across species, leading to their degradation or the modulation of their activity (3). This process involves three main enzymes, namely E1 ubiquitin-activating, E2 ubiquitin-conjugating, and E3 ubiquitin ligases. E3 ligases, including more than 600 varieties in humans, are particularly diverse and specific, and they recognize and bind target proteins for ubiquitination (4). Cullin-RING E3 ligases are multi-subunit complexes composed of the scaffold protein Cullin, an adaptor protein, and a substrate-recognition module (5). The Cullin family and its diverse members play crucial roles in various biological processes, including cell proliferation and response to oxidative stress (6).

Accumulating evidence suggests that diverse viruses can exploit the host UPS to facilitate their replication by targeting antiviral proteins for degradation. For example, the interaction between the human immunodeficiency virus-1 (HIV-1) and the UPS has been extensively studied. The HIV-1 Vif protein promotes the degradation of APOBEC3G, a host factor with intrinsic antiviral activity, via a Cullin 5-containing E3 ligase complex (7–10). In addition, HIV-2 Vpx hijacks host CRL4(DCAF1) E3 ligase to downregulate the expression of antiviral factor SAMHD1, thereby facilitating intrinsic immune evasion (11–13). Moreover, other viruses, such as the influenza A virus, hepatitis B virus, Zika virus, and human papillomavirus, also manipulate the UPS for their benefit (14–18).

In this study, we uncovered a unique interaction between EV-D68 and the host UPS, specifically focusing on the Cullin 3-based E3 ligase. Contrary to the established paradigm in which viruses exploit the UPS to degrade host antiviral factors, our findings demonstrate that Cullin 3-mediated ubiquitination restricts EV-D68 replication by triggering degradation of the viral structural protein VP1. Moreover, EV-D68 was observed to encode a 3C protease capable of cleaving Cullin 3, thereby neutralizing the defense mechanism based on ubiquitin–proteasome degradation. These results enhance our understanding of EV-D68 pathogenesis and reveal an ongoing arms race between the host and virus, wherein the host UPS actively participates in antiviral defense while enteroviruses have evolved strategies to counteract this defensive response.

## RESULTS

### Host Cullin 3 protects against EV-D68 infection

**Fig 1:**
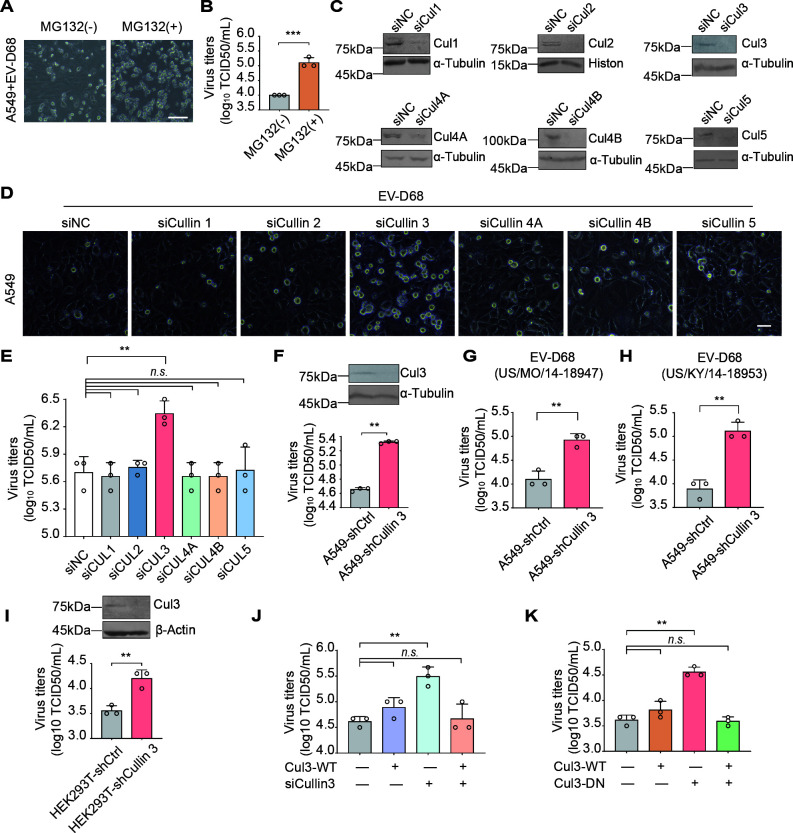
Cullin 3, a scaffold protein for Cullin-RING E3 ligases, promotes EV-D68 replication. (**A**) CPEs in EV-D68-infected A549 cells. Cells were treated with MG132 (10 μΜ) or DMSO 36 h after infection with the EV-D68 virus (Fermon, MOI = 0.01). CPEs were observed 48 h post-infection. (**B**) Titers of progeny virions. Supernatants were gathered 48 h post-infection, and viral titers were measured via a standard plaque assay. Data are expressed as the mean ± standard deviation (SD). ***P < 0.001. (**C**) Validation of Cullin family member knockdown efficiency using immunoblotting. (**D**) CPEs after the Cullin family member knockdown in A549 cells. A549 cells pre-transfected with siRNAs were infected with EV-D68 (Fermon, MOI = 0.01). CPEs were observed 48 h post-infection. (**E**) Viral titers of progeny virions in Cullin family member-knockdown A549 cells. Viral titers were determined via a standard plaque assay. Data are expressed as the mean ± SD. **P < 0.01. *n.s*., not significant. (**F through H**) Viral titers in stable Cullin 3-silenced A549 cells. A549 cells were infected with the EV-D68 prototype Fermon (2014) and isolated US/MO/14-18947 (MO) and US/KY/14-18953 (KY) at an MOI of 0.01. Viral titers were determined using a standard plaque assay 48 h post-infection. Data are expressed as the mean ± SD. **P < 0.01. (**I**) Viral titers in stable Cullin 3-knockdown HEK293T cells. Stable Cullin 3-knockdown or control HEK293T cells were infected with EV-D68 (Fermon, MOI = 0.01). CPEs were observed 48 h post-infection. Data are expressed as the mean ± SD. **P < 0.01. (**J, K**) Viral titers in Cullin 3-inhibited HEK293T cells. HEK293T cells were transfected with siRNAs or dominant-negative Cullin 3. Wild-type Cullin 3 or an empty vector was co-transfected. Cells of all groups were infected with EV-D68 (Fermon, MOI = 0.01) 24 h post-transfection. Viral titers were determined 48 h post-infection. Data are expressed as the mean ± SD. **P < 0.01. *n.s*., not significant.

Various viruses exploit the host UPS to degrade intrinsic antiviral restriction factors (11, 19–21). Consequently, the inhibition of proteasomal degradation is regarded as a promising antiviral strategy and has emerged as a focal point of antiviral research. Based on this premise, we investigated the effect of the proteasome inhibitor MG132 on EV-D68 replication. Contrary to our expectations, MG132 treatment significantly exacerbated the cytopathic effects (CPEs) induced by EV-D68 infection compared to those observed in the dimethyl sulfoxide (DMSO) control group (Fig. 1A), and markedly increased the yield of progeny viruses following the EV-D68 infection (Fig. 1B).

As essential scaffold proteins of intracellular E3 ubiquitin ligases, Cullin family proteins play a critical role in the UPS. To investigate their involvement in EV-D68 replication, we performed an unbiased transient knockdown screen for Cullin family members using the respiratory cell line A549 (Fig. 1C). Only the downregulation of Cullin 3 expression significantly enhanced both the CPEs induced by EV-D68 (Fig. 1D) and the production of progeny viruses (Fig. 1E), whereas other Cullin family members (including Cullin 1, Cullin 2, Cullin 4A, Cullin 4B, and Cullin 5) had no significant effect (Fig. 1D and E). To validate these findings, we established a stable Cullin 3-knockdown A549 cell line. Compared to that in the control group, Cullin 3 silencing markedly increased the viral titer in the supernatant post-infection (Fig. 1F).

Furthermore, we examined the effects of Cullin 3 knockdown on two isolated strains of circulating EV-D68 (MO: US/MO/14-18947 and KY: US/KY/14-18953). Here, Cullin 3 knockdown significantly enhanced viral replication (Fig. 1G and H). To facilitate subsequent mechanistic studies, we evaluated the effect of Cullin 3 on viral replication in HEK293T cells, which are permissive to EV-D68 infection. We noted that Cullin 3 knockdown markedly increased viral replication (Fig. 1I). Complementation experiments further demonstrated that the exogenous expression of Cullin 3 restored the inhibitory effect on EV-D68 replication in Cullin 3 knockdown cells (Fig. 1J). In addition, the overexpression of a dominant-negative mutant, specifically a truncated segment of Cullin 3 (1–418 aa), that inhibits the function of endogenous Cullin 3 confirmed its role in promoting viral replication (Fig. 1K). These findings provide strong evidence that Cullin 3 acts as a critical natural antagonist of EV-D68 replication in host cells.

### Cullin 3 specifically promotes the degradation of EV-D68 VP1 proteins

**Fig 2:**
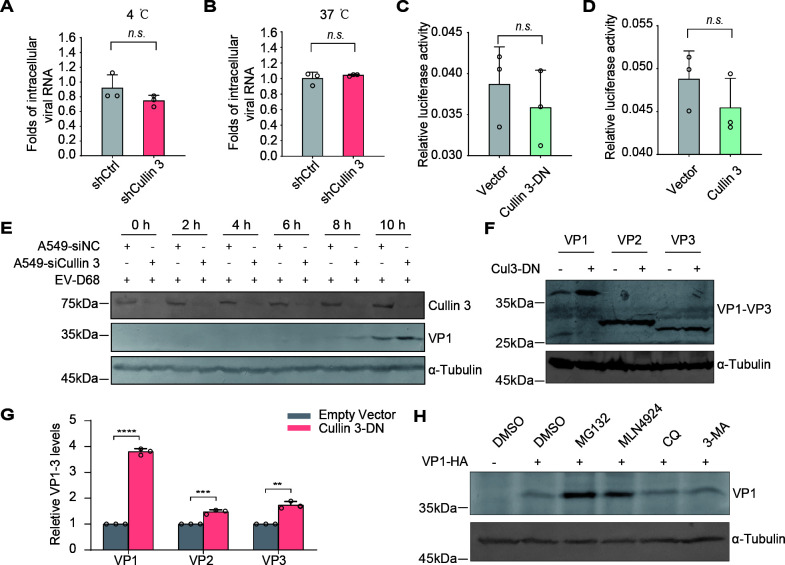
Cullin 3 negatively regulates EV-D68 VP1 protein level. (**A and B**) Virus attachment and entry assays. Stable Cullin 3-knockdown A549 cells were incubated with EV-D68 (Fermon, MOI = 1) at 4 ℃ or 37 ℃ for 2 h, and cells were washed with DMEM to remove unbound viruses. Viral RNA was quantified using qRT-PCR. Data are expressed as the mean ± SD. *n.s*., not significant. (**C and D**) EV-D68 5′ UTR activity. Plasmids with the EV-D68 5′ UTR were transfected into HEK293T cells, along with dominant-negative Cullin 3 (**C**) or wild-type Cullin 3 (**D**) plasmids. Control groups were transfected with a control vector. Renilla luciferase activity served as an internal control. The activities of Firefly and Renilla luciferases were measured 48 h post-transfection. The bar graph represents the ratio of Firefly and Renilla luciferase. Data are expressed as the mean ± SD. *n.s*., not significant. (**E**) EV-D68 VP1 levels in Cullin 3-knockdown A549 cells. A549 cells pre-transfected with the indicated siRNAs were infected with EV-D68 (Fermon, MOI = 0.05). Cells were harvested 2, 4, 6, 8, and 10 h after infection. VP1 expression levels were detected using an immunoblotting assay. (**F**) VP1–VP3 protein levels in Cullin 3-inhibited HEK293T cells. HEK293T cells were transfected with dominant-negative Cullin 3 or control vectors and VP1–VP3 plasmids. VP1–VP3 levels were measured 48 h after transfection. (**G**) The abundance of VP1–VP3 was quantified using ImageJ software. Data are expressed as the mean ± SD. ****P < 0.0001. ***P < 0.001. **P < 0.01. (**H**) Immunoblot analysis of VP1 level. HEK293T cells pre-transfected with the VP1 plasmids were treated with MG132 (10 μΜ), MLN4924 (2 μΜ), CQ (50 μΜ), or 3-MA (5 mΜ). The VP1 levels were measured 12 h after treatment.

Subsequently, we conducted a comprehensive investigation of the mechanism by which Cullin 3 exerts its antiviral effects against EV-D68. Initially, viral attachment and entry assays were performed to exclude any influence of Cullin 3 silencing on the ability of the virus to adhere to the host cell surface and invade cells (Fig. 2A and B). Further validation demonstrated that modulating Cullin 3 expression did not significantly affect the protein translation capability mediated by the viral 5′ untranslated region (UTR) (Fig. 2C and D). However, at the single-round replication time point, as the expression level of Cullin 3 decreased, expression of the viral structural protein VP1 markedly increased (Fig. 2E). At 8 h post-EV-D68 infection, when VP1 was undetectable via western blotting in the control group, VP1 protein expression was detectable in the Cullin 3-knockdown group (Fig. 2E). We further examined the effects of Cullin 3 on the expressions of other EV-D68 viral structural proteins. Cullin 3 selectively influenced the accumulation of the VP1 protein but had no significant effect on the expression levels of VP2 and VP3 (Fig. 2F and G), suggesting that it specifically regulates the stability of the viral structural protein VP1.

Considering that Cullin 3 primarily functions by mediating protein ubiquitination and subsequent proteasomal degradation under normal physiological conditions, we treated the cells with two inhibitors, the proteasome inhibitor MG132 and the neddylation inhibitor MLN4924. Previous studies have shown that neddylation is essential for the ubiquitination activity of Cullin-RING E3 ligases (2, 22, 23). Here, treatment with MG132 or MLN4924 significantly enhanced VP1 accumulation, whereas the autophagy inhibitors CQ and 3-MA had no effect (Fig. 2H). Hence, Cullin 3-mediated ubiquitination selectively induces proteasome-dependent degradation of the EV-D68 structural protein VP1.

### Cullin 3 interacts with EV-D68 VP1 and promotes its ubiquitination

**Fig 3:**
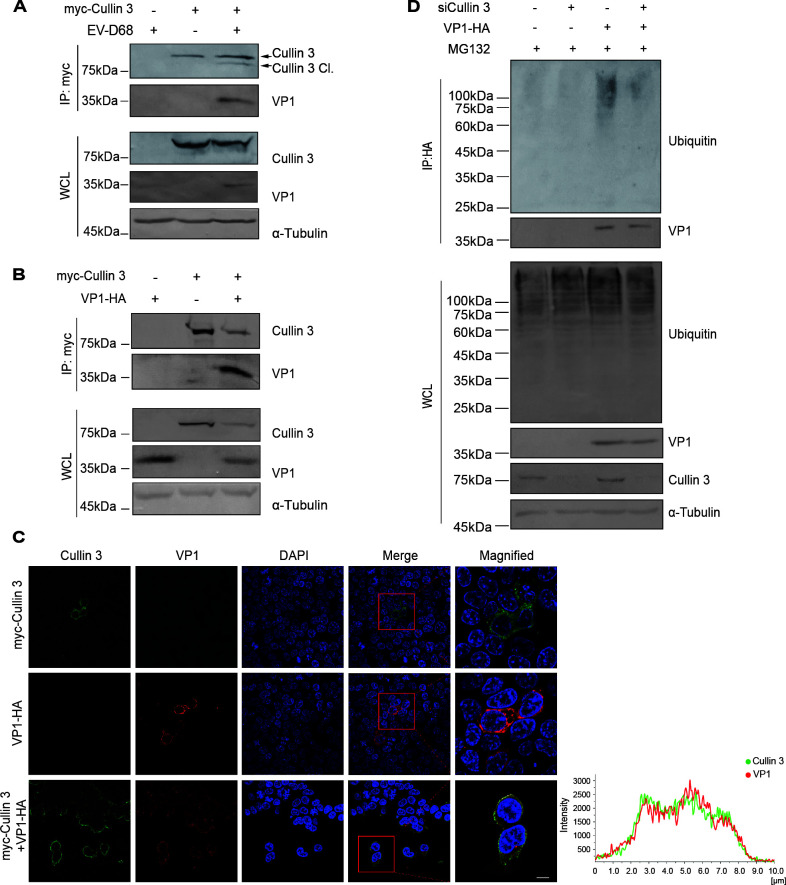
Cullin 3 promotes EV-D68 VP1 ubiquitination. (**A**) Analysis of VP1 in Cullin 3-pull-down precipitates. HEK293T cells pre-transfected with Cullin 3 or empty control constructs were infected with EV-D68 (Fermon, MOI = 0.01). Cells were harvested 48 h post-infection and analyzed via co-IP and immunoblotting. (**B**) Analysis of VP1 in Cullin 3-pull-down precipitates. HEK293T cells pre-transfected with Cullin 3 or empty control constructs, along with VP1-expressing vectors, were harvested 48 h post-transfection and analyzed using co-IP and immunoblotting. (**C**) Subcellular localization of Cullin 3 and VP1. HEK293T cells were transfected with Cullin 3 and VP1 plasmids for 48 h. The cells were then analyzed by performing immunofluorescence and confocal microscopy. Scale bar, 10 µm. (**D**) VP1 ubiquitination analysis. HEK293T cells pre-transfected with siRNAs were transfected with EV-D68 VP1 or empty control vectors. Cells were treated with MG132 (10 μΜ) for 12 h before harvesting and lysis.

To serve as substrates for ubiquitination, target proteins must be recognized by Cullin-RING E3 ubiquitin ligases, forming a complex that catalyzes the transfer of ubiquitin molecules (24, 25). Therefore, we investigated the interaction between the Cullin 3 protein and viral protein VP1. In EV-D68-infected cells, we conducted a co-immunoprecipitation (co-IP) assay by purifying myc-tagged Cullin 3 proteins and observed that the VP1 protein expressed by EV-D68 was effectively enriched (Fig. 3A). No enrichment of VP1 was observed in the control group infected with EV-D68 alone, indicating a specific interaction between Cullin 3 and VP1 (Fig. 3A). Furthermore, we confirmed the direct interaction between Cullin 3 and VP1 using co-IP experiments based on the exogenous expression of myc-Cullin 3 and HA-tagged VP1 proteins (Fig. 3B). In addition, consistent with the co-IP results, we observed colocalization of Cullin 3 and EV-D68 VP1 in HEK293T cells (Fig. 3C).

To explore the regulatory effect of Cullin 3 on the VP1 ubiquitination level, we transfected VP1 expression plasmids or empty vectors into Cullin 3-knockdown HEK293T cells and control cells. After 48 h, co-IP experiments revealed the significant ubiquitination of VP1 in normal cells, whereas Cullin 3 silencing markedly reduced the VP1 ubiquitination level (Fig. 3D). This result corroborates previous observations that Cullin 3 knockdown inhibits the proteasomal degradation of VP1, further supporting the conclusion that Cullin 3 mediates the ubiquitination and degradation of VP1.

### EV-D68 3C triggers the cleavage of Cullin 3 proteins

**Fig 4:**
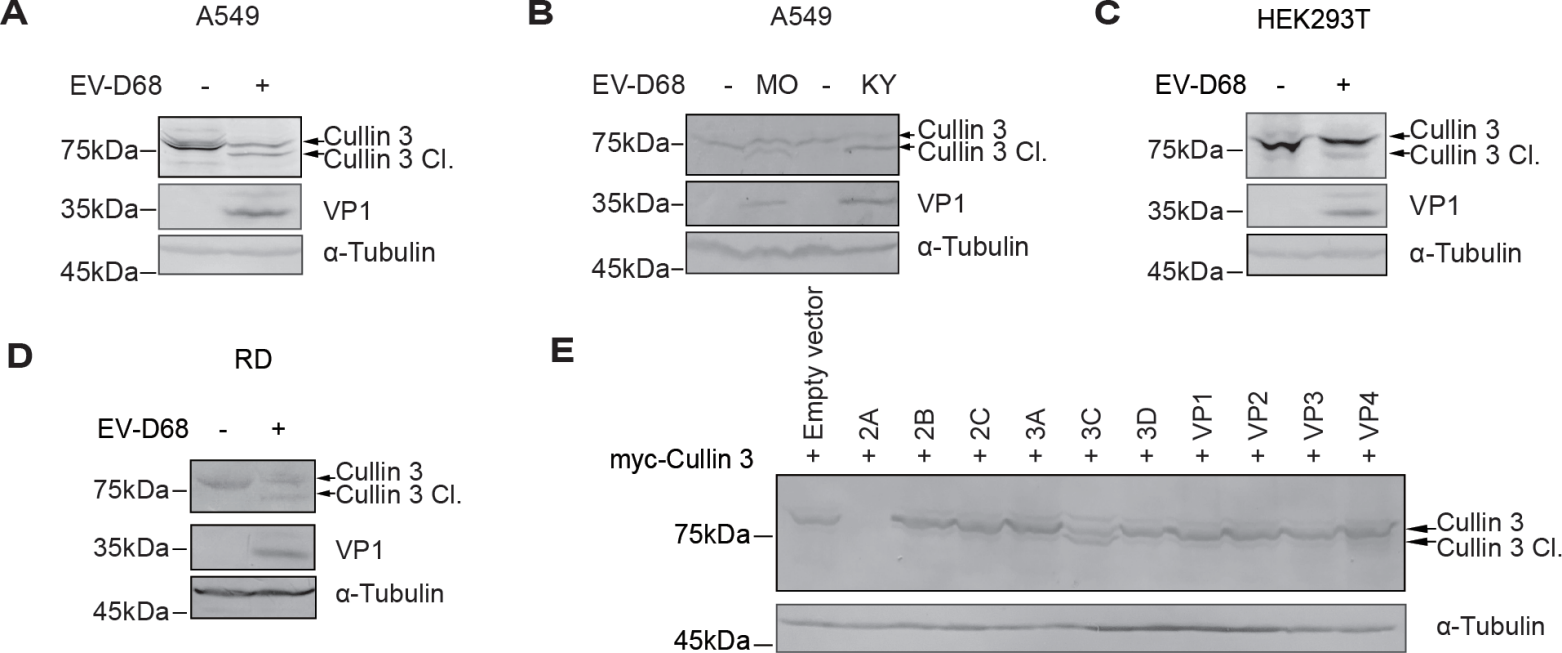
EV-D68 infection triggers the cleavage of Cullin 3 via the enzymatic activity of 3C. (**A, B**) Analysis of the host Cullin 3 level. A549 cells were infected with the EV-D68 prototype Fermon (2014) (**A**) or the isolated US/MO/14-18947 (MO) and US/KY/14-18953 (KY) (**B**) at an MOI of 0.01. Cullin 3 was detected via immunoblotting 48 h post-infection. (**C and D**) Immunoblotting analysis of Cullin 3. HEK293T and RD cells were infected with EV-D68 (Fermon, MOI = 0.01). Cullin 3 was detected 48 h post-infection. (**E**) Screening of Cullin 3 cleavage by EV-D68-encoded proteins. HEK293T cells were transfected with myc-tagged Cullin 3-expressing vectors and indicated EV-D68-encoded proteins. Cell lysates were analyzed 48 h post-transfection.

We observed an intriguing phenomenon wherein EV-D68 infection was associated with the appearance of a lower-molecular-weight Cullin 3 band in immunoblotting assays (Fig. 3A). By contrast, the expression of viral VP1 alone did not result in the detection of this additional Cullin 3 band (Fig. 3B). We therefore hypothesized that EV-D68 might mitigate the inhibitory effects of Cullin 3 on viral infection through a reverse regulatory mechanism targeting Cullin 3. To this end, we examined the effect of different EV-D68 strains (including the prototype strain Fermon and the epidemic strains MO and KY) on Cullin 3 protein expression in A549 respiratory cells. Immunoblotting results demonstrated that these viral infections significantly induced the appearance of additional Cullin 3 bands (Fig. 4A and B). Moreover, Cullin 3 cleavage induced by EV-D68 infection occurred in a cell type-independent manner (Fig. 4C and D). To further elucidate the changes in Cullin 3 protein expression caused by viral infection, we conducted a systematic analysis of the effects of various viral proteins on Cullin 3 expression and observed that the expression of the EV-D68-encoded 3C protease alone induced the appearance of the additional Cullin 3 bands observed during viral infection (Fig. 4E).

**Fig 5:**
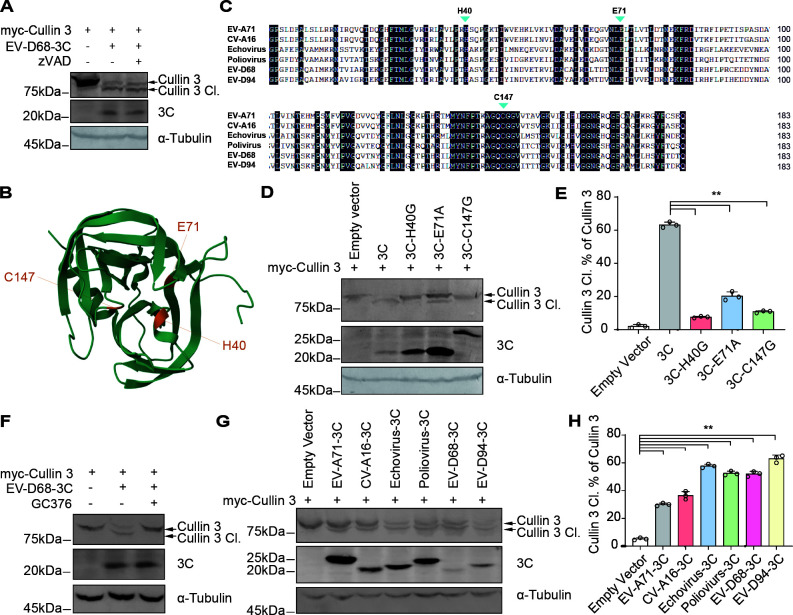
Enterovirus 3C with conserved enzyme activity sites induces Cullin 3 cleavage. (**A**) Cullin 3 cleavage treatment with zVAD. HEK293T cells transfected with Cullin 3 and EV-D68 3C plasmids were cultured in a medium containing Z-VAD-FMK (20 µmol/L) for 48 h. (**B**) Structure of EV-D68 3C protease. (**C**) Sequence alignment of the enteroviral 3C proteases. H40, E71, and C147 indicate the conserved enzyme activity sites. (**D**) Cullin 3 cleavage by 3C protease-defective mutants. (**E**) Relative intensities of cleaved Cullin 3 were quantified using ImageJ software. Data are expressed as the mean ± SD. **P < 0.01. (**F**) Cullin 3 cleavage treatment with GC376. HEK293T cells pre-transfected with Cullin 3 and EV-D68 3C vectors were cultured in a medium containing GC376 (1 µmol/L) for 24 h. (**G**) Cullin 3 cleavage by the indicated enterovirus 3C proteases. (**H**) Relative abundances of cleaved Cullin 3, quantified using ImageJ software. Data are expressed as the mean ± SD. **P < 0.01.

Previous studies have demonstrated that the 3C proteins of enteroviruses can activate host caspases and the associated cell death pathways (26–28). To investigate whether this mechanism influences 3C-mediated Cullin 3 cleavage, we treated the cells with the pan-caspase inhibitor zVAD. zVAD treatment did not alter the extent of Cullin 3 cleavage (Fig. 5A), and therefore, the involvement of caspases in Cullin 3 cleavage was excluded. Subsequently, we examined the role of the intrinsic protease activity of viral 3C in the cleavage of Cullin 3. Structural analysis of the EV-D68 3C protein revealed conserved active sites (H40, E71, and C147), similar to those observed in other enterovirus 3C proteins (Fig. 5B and C). Based on these findings, we generated mutants at these key sites and evaluated their effects on Cullin 3 cleavage (Fig. 5D). All 3C protease activity-deficient mutants (H40G, E71A, and C147G) had a significantly diminished ability to induce Cullin 3 protein cleavage compared to that of the wild-type 3C (Fig. 5E). In addition, treating cells with the 3C-like protease inhibitor GC376 effectively inhibited cleavage of the Cullin 3 protein by the EV-D68 3C protein (Fig. 5F), further confirming that viral 3C cleaves Cullin 3 through its intrinsic protease activity. Finally, we verified that 3C proteins encoded by various enteroviruses (such as EV-A71, CV-A16, Echovirus, poliovirus, EV-D68, and EV-D94) could all cleave Cullin 3 proteins (Fig. 5G and H), indicating that Cullin 3 is a conserved cleavage target of enterovirus 3C proteins.

### 3C induces Cullin 3 cleavage at the Q681 residue

**Fig 6:**
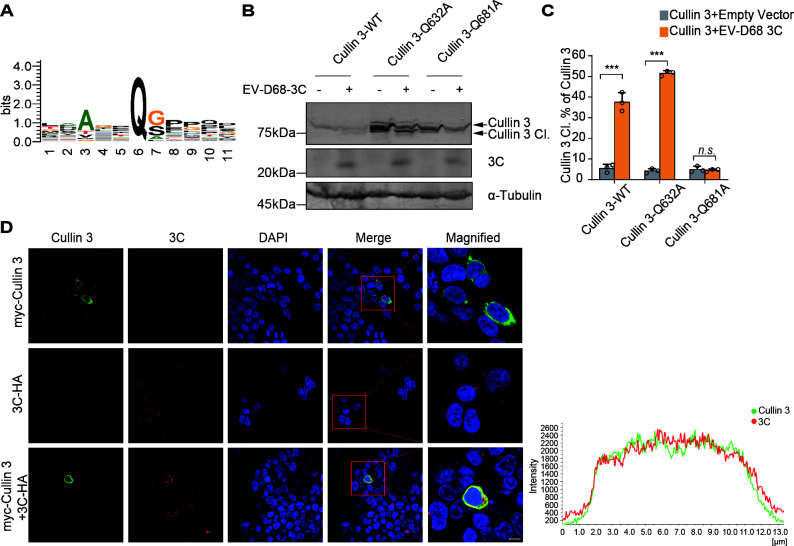
EV-D68 cleaves Cullin 3 at the Q681 residue. (**A**) Sequence logo analysis of the predicted EV-D68 3C protease cleavage site. (**B**) Cullin 3 mutants cleaved by EV-D68 3C. HEK293T cells were transfected with EV-D68 3C and the indicated myc-tagged Cullin 3 plasmids. Cells were harvested 48 h post-transfection. Cullin 3 was detected via immunoblotting. (**C**) Relative densities of cleaved Cullin 3 quantified using ImageJ software. Data are expressed as the mean ± SD. ***P < 0.001. (**D**) Subcellular localization of Cullin 3 and EV-D68 3C. HEK293T cells pre-transfected with Cullin 3 and 3C plasmids were cultured for 48 h. The subcellular localization of these proteins was examined by performing immunofluorescence and confocal microscopy. Scale bar, 10 µm.

Next, we explored the cleavage sites within Cullin 3 proteins and conducted a sequence logo analysis of the known cleavage sites of EV-D68 3C (Fig. 6A). This analysis revealed two conserved motifs in the Cullin 3 (Accession Number: NM_003590.5) sequences. To elucidate the significance of each motif in determining the sensitivity of Cullin 3 to 3C-mediated cleavage, we engineered single-site mutants at positions Q632A and Q681A within Cullin 3. Expression vectors for wild-type Cullin 3 or the specified mutants were co-transfected into cells with plasmids expressing the 3C protease. Immunoblotting assays performed 48 h post-transfection demonstrated that the Q681A mutation significantly impaired EV-D68 3C-mediated cleavage, whereas the Q632A mutation did not affect the sensitivity to 3C compared to that of wild-type Cullin 3 (Fig. 6B and C). We further confirmed the interaction between Cullin 3 and 3C by performing immunofluorescence and confocal microscopy (Fig. 6D). These findings suggest that viral 3C specifically cleaves Cullin 3 at Q681.

### 3C-mediated cleavage impairs Cullin 3-dependent ubiquitination activity

**Fig 7:**
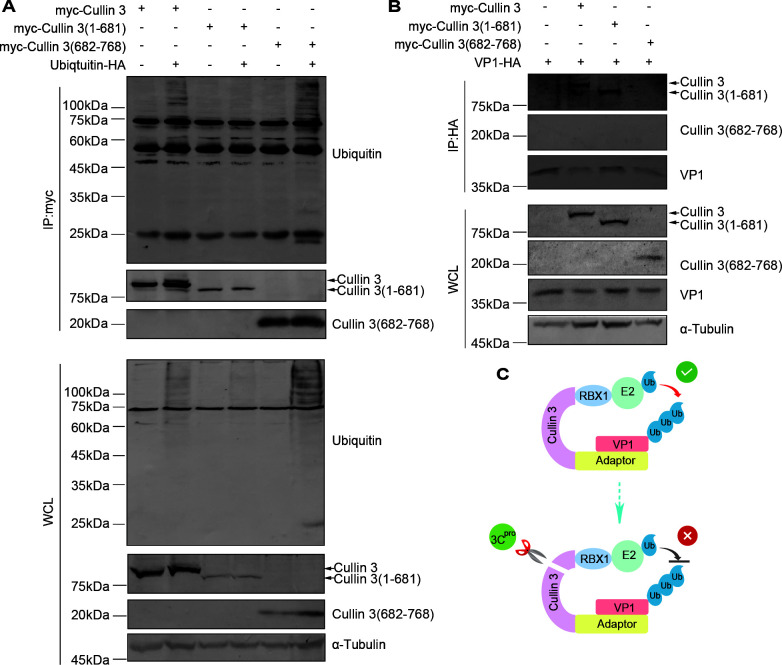
Cullin 3 cleavage disrupts VP1 ubiquitination. (**A**) Immunoblotting analysis of Cullin 3-mediated ubiquitination. HEK293T cells were transfected with the indicated vectors. Whole-cell lysates and myc pull-down products were obtained from HEK293T cell lysates. (**B**) Analysis of Cullin 3 and its cleaved products in VP1-pull-down precipitates. HEK293T cells were transfected with the indicated vectors. Whole-cell lysates and HA pull-down products were analyzed. (**C**) Working model illustrating how VP1 interacts with Cullin 3.

Based on the aforementioned results, it could be deduced that cleavage by the 3C protease results in the fragmentation of Cullin 3 into two distinct segments, Cullin 3 (1–681 aa) and Cullin 3 (682–768 aa). To further elucidate the effect of 3C cleavage on the functionality of Cullin 3, we performed a ubiquitination analysis by transfecting HA-tagged ubiquitination plasmids, along with full-length or truncated Cullin 3 expression plasmids, into HEK293T cells. After a 48 h incubation period, co-IP experiments were conducted. Cullin 3 (682–768 aa) retained its ability to enrich ubiquitination-modified proteins, as with wild-type Cullin 3, whereas Cullin 3 (1–681 aa) entirely lost its ability to bind ubiquitin-bound proteins (Fig. 7A).

Next, we measured the interactions between the 3C cleavage products and EV-D68 VP1. HA-tagged VP1 and full-length or truncated Cullin 3 expression plasmids were transfected into HEK293T cells. Co-IP experiments revealed that Cullin 3 (1–681 aa) maintained its binding affinity for VP1, comparable to that of full-length Cullin 3, whereas Cullin 3 (682–768 aa) exhibited minimal interactions with VP1 (Fig. 7B). In summary, viral protease 3C cleaves and separates the domains of Cullin 3 that are responsible for binding to VP1 and the upstream components of the ubiquitination pathway, thereby disrupting the normal functionality of Cullin 3 (Fig. 7C).

### EV-D68 3C protease inhibits L1 retrotransposition by cleaving Cullin 3

**Fig 8:**
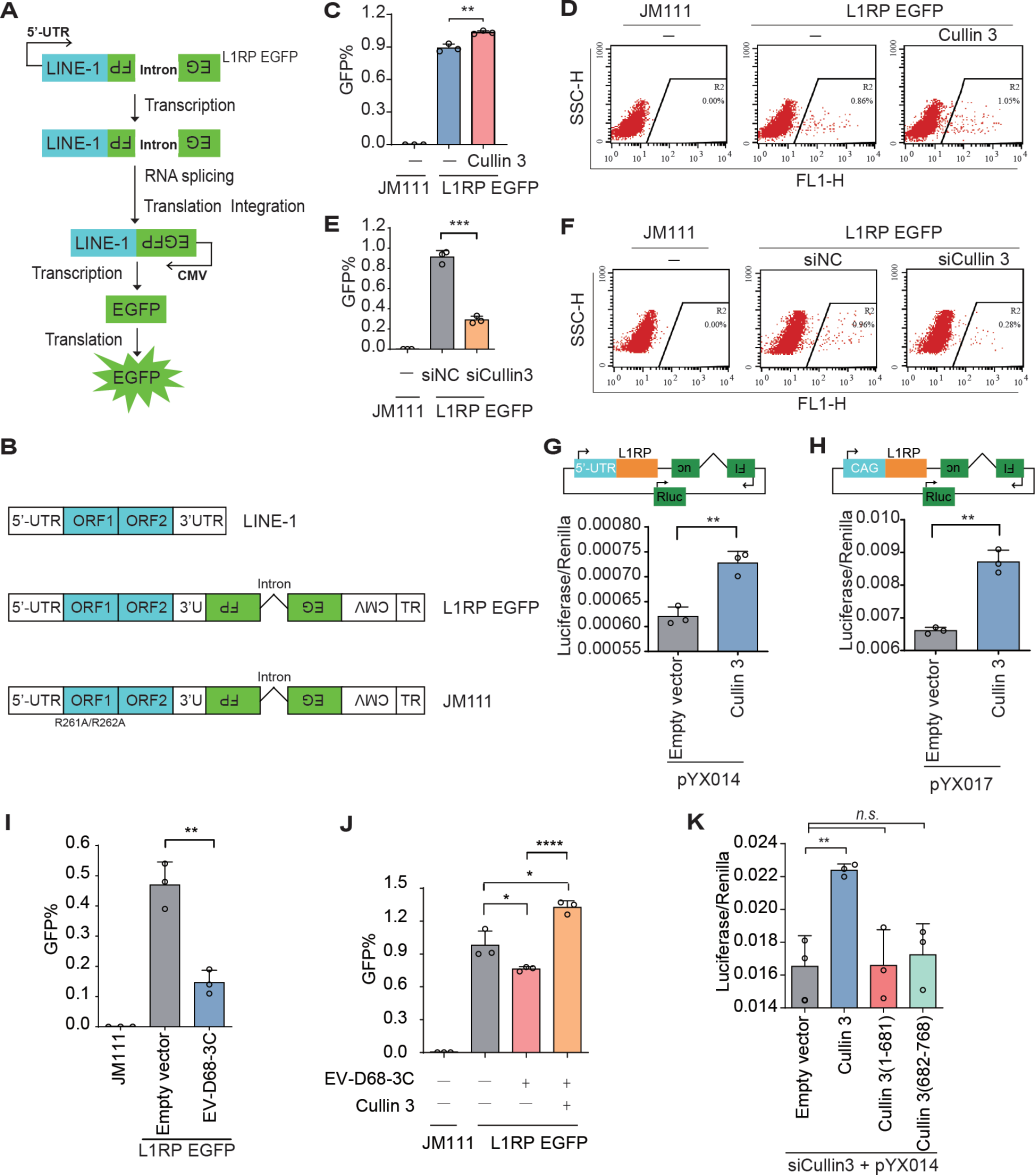
EV-D68 3C suppresses L1 mobilization via Cullin 3 cleavage. (**A**) Schematic representation of EGFP reporter-based retrotransposition assay. A positive signal can only be detected after L1 has been transcribed, spliced, translated, reverse-transcribed, and integrated into the host genome. (**B**) Schematic of LINE-1 plasmids. (**C, D**) L1 retrotransposition mediated by Cullin 3 overexpression, as determined via flow cytometry. HEK293T cells were transfected with L1RP EGFP plasmids, along with Cullin 3 or an empty vector. Transfected cells were selected with puromycin (3 µg/mL for 2 days) 48 h post-transfection. The percentage of GFP (+) cells was measured using flow cytometry. The data are expressed as the mean ± SD. **P < 0.01. (**E, F**) L1 mobility following Cullin 3 knockdown based on flow cytometry. Data are expressed as the mean ± SD. ***P < 0.001. (**G, H**) L1 mobility was determined using a dual luciferase assay. HEK293T cells were transfected with pYX014 or pYX017 plasmids, and L1 mobility was measured using a dual-luciferase assay after 4 days. Data are expressed as the mean ± SD. **P < 0.01. (**I**) L1 mobilization mediated by the EV-D68 3C protease. (**J**) L1 transposition in EV-D68 3C-overexpressing HEK293T cells. HEK293T cells were transfected with L1RP EGFP plasmids, along with EV-D68 3C or an empty vector. Cullin 3 or an empty vector was co-transfected. L1 mobility was measured using flow cytometry after 4 days. The data are expressed as the mean ± SD. *P < 0.1. ****P < 0.0001. (**K**) L1 mobility in Cullin 3-knockdown HEK293T cells. HEK293T cells were transfected with siCullin 3 and pYX014. Cullin 3, Cullin 3 (1–681 aa), Cullin 3 (682–768 aa), or an empty vector were co-transfected. L1 mobility was measured using a dual-luciferase assay after 4 days. The data are expressed as the mean ± SD. **P < 0.01. *n.s*., not significant.

Our previous studies demonstrated that Cullin 3 plays a crucial role in the activity of the human retrotransposon LINE-1 (L1) (29). Increasing evidence indicates that intracellular nucleic acid molecules generated during L1 transposition can activate intrinsic antiviral immune responses (30, 31). Hence, we investigated whether the viral protease 3C-mediated cleavage of Cullin 3 affects host retrotransposition events. We assessed L1 mobility using a well-established L1-EGFP reporter system. The L1RP EGFP plasmid contains full-length L1 sequences with an EGFP reporter cassette interrupted by introns. The EGFP cassette is driven by the CMV promoter; however, the detection of EGFP signals requires successful intron splicing and L1 integration (Fig. 8A). Plasmid JM111, which contains two point mutations in the L1 ORF1 region and is incompetent for retrotransposition, served as the negative control (Fig. 8B). Using this system, we demonstrated that the overexpression of Cullin 3 significantly enhanced the transposition activity of L1 (Fig. 8Cand D). Conversely, Cullin 3 knockdown suppressed L1 activity (Fig. 8Eand F). To further substantiate these findings, we used L1 dual-luciferase reporter systems (pYX014 and pYX017) to quantitatively evaluate the extent to which Cullin 3 augments L1 transposon activity (Fig. 8G and H).

We measured the effects of 3C on L1 transposition and observed that EV-D68 3C efficiently decreased the transposition capacity of human L1 (Fig. 8I). In addition, the overexpression of Cullin 3 counteracted the inhibitory effect of EV-D68 3C on L1 (Fig. 8J). To further explore the effect of 3C-mediated Cullin 3 cleavage on the enhanced L1 transposition activity mediated by Cullin 3, we reconstituted full-length Cullin 3 and the two fragments generated by 3C cleavage in Cullin 3-knockdown cells. Only full-length Cullin 3 effectively restored L1 transposition activity, whereas both Cullin 3 (1–681 aa) and Cullin 3 (682–768 aa) lost this capability (Fig. 8K). These findings suggest that viral protease 3C impairs the transposition activity of L1 elements through the specific cleavage of Cullin 3.

## DISCUSSION

The UPS has been acknowledged for its pivotal role in the degradation of cellular proteins (6). This study provides novel insights into the interaction between EV-D68 and the host UPS, revealing a previously unrecognized mechanism by which the host actively targets enteroviral structural proteins for degradation. This finding challenges the traditional paradigm that diverse viruses predominantly exploit the UPS to degrade host antiviral factors. Instead, our results demonstrate that Cullin 3, a key component of the Cullin-RING E3 ligase complex, restricts EV-D68 replication by promoting ubiquitination and subsequent degradation of the viral capsid protein VP1.

As a component of the E3 ubiquitin ligase complex, Cullin 3 facilitates the ubiquitin-mediated proteasomal degradation of specific substrates, contributing to maintaining cellular protein homeostasis (6). Furthermore, it negatively regulates several signaling pathways, including NF-κB and Nrf2, thereby influencing cellular immune and oxidative stress responses (32–36). Cullin 3 participates in cell cycle regulation by controlling the ubiquitination and degradation of cell cycle-related proteins that are essential for maintaining proper cell proliferation (37). The dysregulation of Cullin 3 is associated with various diseases, including cancer, diabetes, hypertension, and hyperkalemia, making it a potential therapeutic target (38–41). Our findings suggest that Cullin 3 plays a crucial role in limiting viral replication by specifically targeting VP1 for degradation. This specificity underscores the potential of Cullin 3 to serve as a therapeutic target for enhancing host defense against EV-D68 and other enteroviruses.

Previous studies suggested that the proteasomal pathway is required for effective coxsackievirus replication and that proteasome inhibition restricts coxsackievirus B3 replication in murine cardiomyocytes and reduces myocardial damage in mice (42, 43). Moreover, proteasome inhibitors were shown to reduce viral replication by inhibiting viral RNA transcription and suppressing protein translation (42). In our earlier work, we also observed that treatment with inhibitors of the ubiquitin-like modification, neddylation, could suppress the replication of various enteroviruses (44). However, in this study, proteasome inhibitors enhanced EV-D68 replication. This discrepancy could stem from differences in the cell types or animal systems used in the experiments, as well as variations in drug concentration and treatment duration. Proteasome inhibitors broadly block the protein-degradation processes mediated by both Cullin-RING and non-Cullin-RING ubiquitination pathways. This could potentially suppress the proliferation of the immortalized cell lines commonly used for virus culture, thereby indirectly affecting viral replication capacity. Here, we screened different Cullin proteins and found that Cullin 3-dependent ubiquitination could lead to VP1 protein degradation. Moreover, the KLHL12-based CUL3 E3 ubiquitin ligase complex has been reported to induce the ubiquitination of KHSRP, thereby promoting the inhibition of EV-A71 internal ribosome entry sites-driven viral translation mediated by KHSRP (45). All of these results suggest that proteasome inhibition alone has certain limitations when combating viruses. In addition to their potential toxicity to host cells (both infected and uninfected), they may unintentionally enhance the replication of viruses such as EV-D68.

The ability of EV-D68 to counteract the UPS-mediated defense of the host through its 3C protease activity is a testament to the ongoing arms race between the host and the virus. The cleavage of Cullin 3 at the Q681 residue by the 3C protease represents a strategic mechanism used to effectively abrogate the E3 ligase activity of Cullin 3. This cleavage event separates the VP1-binding domain from the E2-binding domain, thereby preventing Cullin 3 from mediating the ubiquitination and subsequent degradation of VP1. Moreover, conservation of this cleavage mechanism among different enteroviruses, including EV-A71 and poliovirus, highlights the evolutionary pressure on these viruses to evade host defenses.

Cullin 3 functions as the core scaffold protein of the CRL3 E3 ubiquitin ligase complex, assembling with BTB domain adaptors and the RING finger protein RBX1 to mediate substrate ubiquitination (46). It also plays essential roles in maintaining cellular homeostasis through both degradative and non-degradative ubiquitination mechanisms. These include the regulation of cell cycle progression, controlling X chromosome inactivation through ubiquitin-mediated transcriptional silencing, modulating protein subcellular localization and trafficking, facilitating DNA damage repair by recruiting repair factors such as 53BP1 to damage sites, and maintaining oxidative stress responses via the KEAP1–Nrf2 pathway (37, 47–52). Our findings reveal that enteroviral 3C proteases specifically cleave Cullin 3, resulting in its functional inactivation. This effect should be particularly significant for persistent infections caused by pathogens such as Coxsackievirus B, where sustained Cullin 3 dysfunction may lead to severe pathological consequences, including genomic instability due to disrupted cell cycle regulation and DNA replication stress, organelle dysfunction due to impaired protein trafficking, increased genomic mutation susceptibility due to compromised DNA repair mechanisms, and oxidative stress-related pathologies stemming from dysregulated redox homeostasis. Our results suggest that enteroviruses may induce long-term cellular dysregulation by targeting the host ubiquitin–proteasome system through Cullin 3 cleavage. This raises important questions about whether virus-mediated Cullin 3 inactivation could globally disrupt protein homeostasis and contribute to viral pathogenesis, which warrants further investigation for a better understanding of enteroviral disease mechanisms.

As previously discussed, Cullin 3-based E3 ubiquitin ligases have critical regulatory functions in diverse physiological processes. Our recent investigation demonstrated that Cullin 3-mediated ubiquitination is indispensable for sustaining the transposition activity of the intracellular retrotransposon L1 (29). In this study, we further observed that the 3C protease of EV-D68 impairs L1 transposition activity by cleaving and inactivating Cullin 3, thereby revealing the molecular mechanism by which the viral protein 3C inhibits L1 transposition. Previous research indicated that L1 transposon activity is unexpectedly activated during various viral infections (53, 54). In addition, accumulating evidence suggests that the L1 RNA and DNA generated during transposition can trigger an innate immune response detrimental to viral infection and transmission (30, 31). Through our exploration of the interaction between Cullin 3 and the EV-D68 virus, we observed that viral protease 3C counteracts the antiviral effects mediated by Cullin 3 and mitigates the adverse effect of L1 transposition on viral amplification.

By uncovering the role of Cullin 3 as a restriction factor and the counteractive strategy used by the EV-D68 3C protease, we have expanded our understanding of host antiviral mechanisms and viral evasion tactics. Our study provides an understanding of the complex interplay between EV-D68 and the host UPS has broader implications for the development of therapeutic strategies against enteroviruses. Targeting the UPS or inhibiting viral proteases may enhance host defense mechanisms and could be used as a strategy to develop more effective antiviral treatments.

## MATERIALS AND METHODS

### Cells and virus

A549, HEK293T, and RD cells were cultured in Dulbecco’s modified Eagle’s medium (DMEM) supplemented with 10% fetal bovine serum and penicillin-streptomycin (100 g/mL). To generate stable lentivirus-transduced cell lines, HEK293T cells were co-transfected with the shCullin 3 constructs, pMDLg/pRRE (Addgene, 12251), pRSV-Rev (Addgene, 12253), and pCMV-VSV-G (Addgene, 8454). Two days post-transfection, the medium was collected and filtered with a 0.45 µm filter, and the virus particles were concentrated via ultracentrifugation at 28,000 rpm for 2 h over a 20% sucrose cushion. The resulting lentivirus pellet was resuspended in DMEM and stored at –80°C. A549 and HEK293T cells infected with the lentivirus were further subjected to puromycin selection.

The EV-D68 prototype Fermon (VR-1826, ATCC) and isolated US/MO/14-18947 (VR-1823D, ATCC) and US/KY/14-18953 (VR-1825D, ATCC) were propagated in RD cells. Viral supernatants were collected 3 days after infection, followed by three cycles of freezing and thawing. Samples were centrifuged at low speed and then filtered through a 0.22 µm filter. Viral particles were precipitated using 20% sucrose and centrifuged at 28,000 rpm for 120 min using a SW28 rotor (Beckman). The purified viral particles were stored at −80°C.

### Plasmids, siRNA knockdown, and reagents

The human L1 plasmids 99 PUR JM111 EGFP (referred to as JM111) and 99 PUR L1RP EGFP (referred to as L1RP EGFP) were generously provided by Dr H. H. Kazazian Jr. and Dr. Goodier John L. JM111 and L1RP EGFP were created as detailed in previous studies (55–57). The JM111 construct, which harbors two missense mutations in the ORF1 region, served as a negative control in our experiments. In addition, the pYX014 and pYX017 plasmids were kindly provided by Professor Wenfeng An (58, 59). The N-terminal myc-tagged Cullin 3 constructs were a gift from Professor Xiangpeng Dai of the First Hospital of Jilin University (38). shCullin 3 constructs were purchased from Generay Biotech (Shanghai, CN). Furthermore, dominant-negative Cullin 2 (Addgene, 41912), Cullin 4A (Addgene, 41914), Cullin 4B (Addgene, 41915), and Cullin 5 (Addgene, 41916) plasmids were acquired from Addgene. Flag-tagged dominant-negative Cullin 1 and Cullin 3 were purchased from Generay Biotech (Shanghai, CN). The expression plasmids (EV-D68 2A, 2B, 2C, 3A, 3C, 3D, VP1, VP2, VP3, VP4, EV-A71 3C, CV-A16 3C, Echovirus 3C, Poliovirus 3C, and EV-D96 3C) were purchased from Generay Biotech (Shanghai, China). EV-D68 3C mutants (H40G, E71A, and C147G) and Cullin 3 mutants (Q632A and Q681A) were generated via site-specific mutagenesis. The truncated myc-Cullin 3 (1–681) and myc-Cullin 3 (682–768) variants were generated using gene recombination technology with myc-tagged Cullin 3 as the template. The empty vector VR1012 and pcDNA3.1(+) were sourced from our laboratory.

Small interfering RNA (siRNA) oligonucleotides were custom synthesized by RiboBio. The pooled siRNAs were introduced into HEK293T cells at a final concentration of 100 nM using Lipofectamine 3000 (Invitrogen). The following siRNAs were used: si-human-CUL1-001 (5′-GCCCTACGTTAACAGTGTA-3′), si-human-CUL1-002 (5′-GCCCAATCATCCAGTAAAT-3′), si-human-CUL1-003 (5′-GGGTTCGAGTACACCTCTA-3′), si-human-CUL2-001 (5′-GGAGGAAATTGATGGTTGA-3′), si-human-CUL2-002 (5′-GAAGGAAACTTACATGGTT-3′), si-human-CUL2-003 (5′-GCACAATGCCCTTATTCAA-3′), si-human-CUL3-001 (5′-GAAGGAATGTTTAGGGATA-3′), si-human-CUL3-002 (5′-GCACTGCCTTGACAAATCA-3′), si-human-CUL3-003 (5′-CAATGACCGTCTCTTTAAA-3′), si-human-CUL4A-001 (5′-GGAAGAGACTAATTGCTTA-3′), si-human-CUL4A-002 (5′-GCATGTGGATTCAAAGTTA-3′), si-human-CUL4A-003 (5′-CGAAGGACATCATGGTTCA-3′), si-human-CUL4B-001 (5′-GGTGAACACTTAACAGCAA-3′), si-human-CUL4B-002 (5′-CTACCACCGTCTCTAGCTT-3′), si-human-CUL4B-003 (5′-GAAGGAATGTTTAAAGACA-3′), si-human-CUL5-001 (5′-GAATGAAGTTGGTCAATAT-3′), si-human-CUL5-002 (5′-GCTGCAGACTGAATTAGTA-3′), si-human-CUL5-003 (5′-GGACAAAGTTCCTAATGGT-3′), and control nontargeting siRNA (referred to as siNC).

The following reagents were used: MG132 (MedChemExpress, HY-13259), MLN4924 (MedChemExpress, HY-70062), CQ (MedChemExpress, HY-17589A), 3-MA (MedChemExpress, HY-19312), Z-VAD-FMK (MedChemExpress, HY-16658B), and GC376 (SelleckChem S0475). The following antibodies were used: anti-Cullin 1 antibody (Abcam, ab75817), anti-Cullin 2 antibody (Abways, CY1025), anti-Cullin 3 antibody (Proteintech, 11107-1-AP), anti-Cullin 4A antibody (Proteintech, 10693-1-AP), anti-Cullin 4B antibody (Proteintech, 12916-1-AP), anti-Cullin 5 antibody (arigo, ARG59155), anti-EV-D68-VP1 antibody (GeneTex, GTX132313), anti-HA-tag antibody (Thermo Fisher, 71-5500), anti-Ubiquitin antibody (Proteintech, 10201-2-AP), anti-myc-tag antibody (Sigma, M5546), anti-α-Tubulin antibody (GenScript, A01410), and anti-histone H3 antibody (Abcam, ab176842).

### Virus titer assay

Viral titers were determined using an endpoint dilution assay. RD cells were cultured under standard conditions in 96-well plates at a density of 10,000 cells/well. EV-D68 was subjected to a stepwise 10-fold dilution in DMEM supplemented with 1% FBS before being introduced into the cells. The assessment of viral titers relied on the detection of CPEs in RD cells, utilizing a microtitration analysis in line with the Reed–Muench calculation.

### EGFP-based retrotransposition reporter assay

HEK293T cells were cultured in 24-well plates and transfected with the L1RP EGFP vector. Following transfection, the cells were subjected to puromycin selection based on a concentration of 3 µg/mL, 48 h post-transfection. The proportion of GFP-positive cells was quantified using a BD FACSCalibur Flow Cytometer, 96 h post-transfection. The JM111 plasmid, which is characterized by two missense mutations in its ORF1 sequence, served as a control for non-specific signal exclusion based on its background fluorescence. For flow cytometric analysis, a sample-specific threshold of 10,000 single-cell events was gated, and retrotransposition events were analyzed using CellQuest Pro (v.5.2).

### Dual luciferase-based retrotransposition reporter assay

HEK293T cells were cultured in 24-well plates at an initial density of 1 × 10^5^ cells per well and transfected with the pYX014/pYX017 vector on the day after seeding. Subsequently, transfected cells were selected with puromycin at a concentration of 3 µg/mL 24 h post-transfection. Dual-luciferase assays were performed 96 h post-transfection according to the manufacturer’s instructions (Promega). The luminescence of both Firefly and Renilla luciferases was assessed in a single sample using Promega GloMax (Sunnyvale, USA).

### Immunoblotting

Cell membranes were permeabilized with RIPA buffer (1% NP-40, 0.5 M EDTA, 1 M Tris pH 7.8, and 1 M NaCl). The resulting cell lysates were electrophoresed on a 12% sodium dodecyl sulfate-polyacrylamide gel and transferred onto nitrocellulose membranes. The antibodies were used in accordance with the manufacturer’s guidelines. The signal ratios from the immunoblotting analyses were computed using ImageJ software.

### Co-immunoprecipitation

For co-IP experiments, pre-transfected HEK293T cells were collected and washed twice with cold phosphate-buffered saline. The cells expressing the protein of interest were then lysed in a buffer containing 50 mM Tris (pH 7.5), 150 mM NaCl, 0.5% NP40, and a complete protease inhibitor tablet (Roche) at 4°C for 1 h. The lysates were then clarified via centrifugation at 10,000 × *g* for 30 min at 4°C. The lysates were subsequently incubated with either anti-myc magnetic beads (BeyoMag, P2118-0.5 mL) or anti-HA antibody-conjugated agarose beads (Roche, 11815016001) at 4°C overnight. The reaction mixtures were washed six times with a buffer containing 20 mM Tris (pH 7.5), 100 mM NaCl, 0.1 mM EDTA, and 0.05% Tween 20. Bound proteins were eluted using a buffer containing 100 mM glycine–HCl (pH 2.5) and then analyzed via immunoblotting.

### Immunofluorescence and confocal microscopy

Pre-transfected HEK293T cells were cultured on a glass-bottom dish (Nest, 801001) for 48 h. Subsequently, the cells were fixed with 4% paraformaldehyde for 30 min, permeabilized with 0.1% Triton X-100 for 30 min, and blocked using 5% bovine serum albumin for 1 h. Then, the cells were incubated with an anti-HA-tag or anti-myc-tag antibody at 4 ℃ overnight. Next, the cells were incubated with an Alexa Fluor 594-conjugated antibody (Life Technologies/Thermo Fisher Scientific, A-11012) and an Alexa Fluor 488-conjugated antibody (Life Technologies/Thermo Fisher Scientific, A11088) at room temperature for 1 h. The nuclei were stained with 4,6-diamidino-2-phenylindole. Confocal images were obtained using a Nikon laser-scanning confocal microscope.

### Sequence logo analysis

The sequence logo was generated using the WebLogo website (http://weblogo.threeplusone.com/create.cgi) based on the alignment results obtained from MEGA7 (Molecular Evolutionary Genetic Analysis 7). The following cleavage sites were included: EV-D68 self-cleavage sites (60); TFEB, GSDMD, STAT1, and TDP-43 cleavage sites associated with EV-D68 (Fermon) (61–64); OAS3 cleavage site associated with EV-D68 (US/KY/14–18953) (65); TRIF, IRF7, and CD74 cleavage sites associated with a Beijing strain (GenBank accession number KF726085) of EV-D68 (66–68).

### Quantification and statistical analysis

Statistical analyses were performed using GraphPad Prism software (version 8.0.2; GraphPad Software Inc.). Differences between test groups were analyzed using the unpaired Student’s t-test. The statistical significance was set at *P* < 0.05.

## Data Availability

The data that support the findings of this study are available from the corresponding author upon reasonable request.
